# Systemic Inflammatory Burden Is Independently Associated with Anemia After Transcatheter Aortic Valve Implantation in Patients Referred to Cardiac Rehabilitation

**DOI:** 10.3390/biomedicines14071584

**Published:** 2026-07-15

**Authors:** Theodor Constantin Stamate, Radu Sebastian Gavril, Mihai Roca, Raul-Alexandru Jigoranu, Alexandru-Dan Costache, Mihai Stefan Cristian Haba, Georgeta Zugravu, Alexandra Mastaleru, Andra Oancea, Maria Magdalena Leon, Florin Mitu

**Affiliations:** 1 Grigore T. Popa University of Medicine and Pharmacy Iași, 700115 Iași, Romania; stamate.theodor@gmail.com (T.C.S.); roca2m@yahoo.com (M.R.); alexandru.jigoranu@umfiasi.ro (R.-A.J.); adcostache@yahoo.com (A.-D.C.); mihai.haba@umfiasi.ro (M.S.C.H.); alexandra.mastaleru@gmail.com (A.M.); andra.oancea@umfiasi.ro (A.O.); maria.leon@umfiasi.ro (M.M.L.); florin.mitu@umfiasi.ro (F.M.); 2 Clinical Rehabilitation Hospital, 700661 Iași, Romania; zugravu.georgeta@scr.ro; 3 Department of Cardiology, “St. Spiridon” County Clinical Emergency Hospital, 700111 Iași, Romania; 4 Romanian Academy of Medical Sciences, 030167 Bucharest, Romania; 5 Romanian Academy of Scientists, 050045 Bucharest, Romania

**Keywords:** transcatheter aortic valve implantation, anemia, neutrophil-to-lymphocyte ratio, platelet-to-lymphocyte ratio, systemic immune–inflammation index, cardiac rehabilitation, inflammation

## Abstract

**Background:** Anemia is frequently observed in patients undergoing transcatheter aortic valve implantation (TAVI) and may reflect persistent biological vulnerability in the post-procedural period. The relationship between systemic immune–inflammatory burden and anemia at entry into structured cardiac rehabilitation (CR) after TAVI remains insufficiently explored. **Methods:** This retrospective observational study included 80 consecutive patients referred to CR after successful TAVI. Anemia was defined by World Health Organization (WHO) criteria (hemoglobin <13 g/dL in men; <12 g/dL in women). Systemic inflammatory burden was assessed using C-reactive protein (CRP), neutrophil-to-lymphocyte ratio (NLR), platelet-to-lymphocyte ratio (PLR), monocyte-to-lymphocyte ratio (MLR), and the systemic immune–inflammation index (SII = platelet × neutrophil/lymphocyte). Multivariable logistic regression, adjusted for age, sex, body mass index (BMI), type 2 diabetes mellitus, and estimated glomerular filtration rate (eGFR), was used to evaluate independent associations with anemia. **Results:** Anemia was present in 67.5% of patients at CR entry. Compared with non-anemic patients, anemic patients exhibited significantly higher CRP (4.25 vs. 2.58 mg/L; *p* = 0.014), NLR (2.86 vs. 2.34; *p* = 0.004), PLR (132.33 vs. 102.13; *p* = 0.003), and SII (566.01 vs. 448.30; *p* = 0.011). In multivariable models, NLR (OR 4.09; 95% CI 1.50–11.19; *p* = 0.006), PLR (OR 4.89; 95% CI 1.41–17.02; *p* = 0.013), SII (OR 4.96; 95% CI 1.35–18.14; *p* = 0.016), and CRP (OR 2.17; 95% CI 1.07–4.39; *p* = 0.032) were independently associated with anemia. Area under the ROC curve (AUC) was 0.70 for NLR and PLR, 0.68 for SII, and 0.67 for CRP. **Conclusions:** Systemic inflammatory burden is independently associated with anemia at the time of entry into CR facility after TAVI. These findings support an integrated approach to patient evaluation and provide a basis for future mechanistic and interventional studies.

## 1. Introduction

Transcatheter aortic valve implantation (TAVI) has become the standard therapeutic approach for patients with severe symptomatic aortic stenosis who are at high or intermediate surgical risk [1,2]. By effectively relieving valvular obstruction, TAVI leads to rapid hemodynamic improvement and symptomatic relief [1,2]. However, despite correction of the mechanical component of the disease, many patients remain clinically and biologically vulnerable in the early post-procedural period [3,4].

Persistent systemic inflammation after TAVI has been increasingly recognized as a relevant pathophysiological phenomenon [3,4]. The procedure itself, underlying comorbidities, advanced age, and chronic cardiovascular remodeling all contribute to sustained immune activation [3,4]. A systemic inflammatory response syndrome (SIRS) develops in approximately 56% of patients in the early post-TAVI period and has been associated with adverse short- and long-term outcomes [3,4]. Beyond classical inflammatory markers such as C-reactive protein (CRP), composite indices derived from peripheral blood cell counts—such as the neutrophil-to-lymphocyte ratio (NLR), platelet-to-lymphocyte ratio (PLR), and the systemic immune–inflammation index (SII)—have emerged as accessible and potentially more integrative indicators of immune–inflammatory balance [5,6,7]. SII, calculated as the product of platelet and neutrophil counts divided by lymphocyte count, integrates three circulating immune-cell lineages into a single parameter and has been demonstrated to be independently associated with major adverse cardiac events and short-term mortality in TAVI patients [7,8,9,10].

Anemia is another frequent finding in patients undergoing TAVI, with reported prevalence ranging from 39% to 72% depending on the population, definition, and timing of assessment [11,12,13]. Its etiology is multifactorial, including procedural blood loss, chronic kidney disease, nutritional deficiencies, and chronic disease-related mechanisms [13,14,15,16]. Inflammatory activation may play a central role by impairing iron metabolism and erythropoiesis through hepcidin-mediated pathways [14,15]. Nevertheless, the relationship between systemic inflammatory burden and anemia in patients entering structured cardiac rehabilitation after TAVI remains insufficiently explored.

Despite growing evidence supporting the prognostic value of composite inflammatory indices in cardiovascular diseases and specifically in TAVI patients [5,6,7,8,9,10,17], current research has primarily focused on their association with mortality, procedural complications, and major adverse cardiovascular events. While anemia is highly prevalent in this population and has been linked to worse outcomes [11,12,13], existing studies have largely evaluated it as an isolated variable rather than in conjunction with systemic inflammatory burden. We did not identify prior studies specifically evaluating the relationship between composite immune–inflammatory indices and anemia at the specific clinical timepoint of cardiac rehabilitation entry after TAVI. This gap is clinically relevant, as it represents a transitional period characterized by persistent biological vulnerability [18,19].

Therefore, the aim of this study was to evaluate the prevalence of anemia in patients entering CR programmes after TAVI and to investigate the independent association between systemic immune–inflammatory indices—including SII, NLR, PLR, MLR, and CRP—and anemia after adjustment for relevant clinical and metabolic confounders.

## 2. Materials and Methods

**Figure 1:**
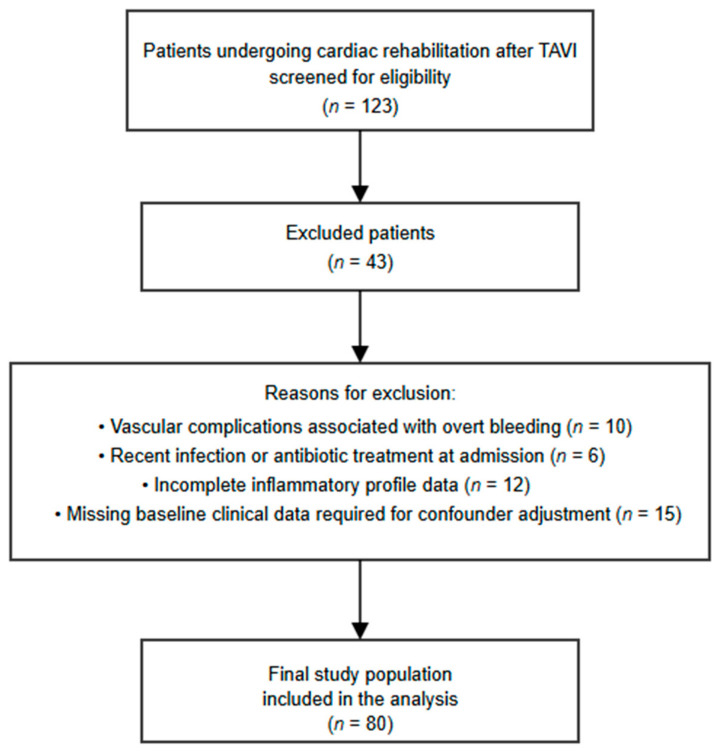
Flow chart diagram of patients hospitalized in the Cardiovascular Rehabilitation Clinic Unit between January 2023 and September 2025.

This retrospective observational study included all patients referred to cardiac rehabilitation in the Cardiovascular Unit of the Clinical Rehabilitation Hospital in Iasi, between January 2023 and September 2025, following successful TAVI performed at a tertiary cardiovascular center. All patients were transferred from the cardiac surgery department after procedural stabilization and enrolled in a structured in-hospital CR program. Per institutional protocol, transfer to the CR unit occurred following confirmed hemodynamic stabilization, typically within 5–14 days post-procedure. Demographic, clinical, electrocardiographic, echocardiographic, and laboratory data were retrospectively extracted from institutional electronic medical records. Only patients with complete hematologic and inflammatory data at baseline were included. To limit potential confounders affecting inflammatory status and post-procedural anemia, patients with a recent infection or ongoing antibiotic therapy at the time of admission were excluded. Moreover, patients were excluded if they developed minor, major, or life-threatening periprocedural bleeding according to VARC-2 criteria, presented with any overt access-site or non-access-site vascular complications, or required red blood cell transfusion during the index TAVI hospitalization (Figure 1).

Anemia was defined according to WHO criteria as hemoglobin <13 g/dL in men and <12 g/dL in women [20]. Based on this definition, patients were categorized into anemic and non-anemic groups.

Systemic immune–inflammatory burden was assessed at admission to CR facility using routinely available laboratory parameters: CRP, NLR, PLR, MLR, and SII. The SII was calculated as SII = platelet count × neutrophil count / lymphocyte count. All measurements were obtained from standard venous blood samples collected as part of routine clinical evaluation at CR entry.

Potential confounders were selected based on biological plausibility as established determinants of both systemic inflammation and anemia in the cardiovascular literature. The five covariates retained in the adjusted models were age, sex, BMI, type 2 diabetes mellitus, and eGFR.

Continuous variables were tested for normality using the Shapiro–Wilk test and are presented as median with interquartile width (IQW). Categorical variables are presented as counts and percentages. Comparisons between anemic and non-anemic patients were performed using the Mann–Whitney U test for continuous variables and the chi-square test or Fisher’s exact test for categorical variables.

Multivariable logistic regression analyses were conducted separately for each inflammatory marker to evaluate independent associations with anemia. Continuous predictors were standardized and expressed per one standard deviation increase. Adjusted odds ratios (ORs) with 95% confidence intervals (CIs) were reported. Given the sample size (*n* = 80; 54 anemia events) and the intercorrelated nature of composite inflammatory indices, separate multivariable models were constructed for each marker rather than a single combined model, in order to preserve adequate events-per-variable ratios (EPV = 9 per model) and avoid multicollinearity. Multicollinearity diagnostics indicated no significant correlations among independent variables (variance inflation factor < 5 for all predictors). Model calibration was assessed using the Hosmer–Lemeshow goodness-of-fit test; adequate calibration was confirmed for all models (all *p* > 0.05: NLR *p* = 0.568, PLR *p* = 0.648, SII *p* = 0.976, CRP *p* = 0.672, MLR *p* = 0.810). Internal validation was performed using 1000-iteration bootstrap resampling to estimate optimism-corrected AUC values for each multivariable model; mean optimism ranged from 0.052 to 0.063, with bootstrap-corrected AUC values of 0.771 for NLR, 0.737 for PLR, 0.752 for SII, 0.732 for CRP, and 0.719 for MLR, indicating modest overfitting and acceptable model stability given the sample size. A two-sided *p*-value < 0.05 was considered statistically significant. To account for multiple comparisons across the five inflammatory markers, the Benjamini–Hochberg false discovery rate (FDR) procedure was applied. Given that these indices represent intercorrelated measures of a shared immune–inflammatory axis, FDR control was preferred over methods assuming independence between tests (e.g., Bonferroni), which would substantially overcorrect for non-independent comparisons. Associations were considered to survive correction at an FDR threshold of *q* < 0.05. Data analysis was performed using SPSS version 20.0 (IBM Corp., Armonk, NY, USA); analytical outputs were cross-checked for consistency.

The study was conducted in accordance with the Declaration of Helsinki and received ethical approval from two institutional review boards: the Research Ethics Committee of “Grigore T. Popa” University of Medicine and Pharmacy, Iași, Romania (approval no. 742/22.03.2026) and the Ethics Committee of Clinical Rehabilitation Hospital, Iași, Romania (approval no. 06/20.05.2025).

## 3. Results

A total of 80 patients were included in the analysis. At admission to cardiac rehabilitation programme, anemia according to WHO criteria was present in 67.5% (*n* = 54) of patients. No significant differences were observed between anemic and non-anemic patients regarding age or sex distribution. Patients with anemia had a significantly lower BMI and a higher prevalence of type 2 diabetes mellitus. Systolic and diastolic blood pressure values were significantly lower in the anemic group, whereas heart rate and eGFR did not differ between groups. LVEF categories and the prevalence of atrial fibrillation were comparable between groups. The clinical and laboratory characteristics of the study population, stratified by anemia status, are summarized in Table 1.

Regarding inflammatory markers, median CRP values were significantly higher in anemic patients compared with non-anemic patients (4.25 vs. 2.58 mg/L; *p* = 0.014). Similarly, NLR (2.86 vs. 2.34; *p* = 0.004), PLR (132.33 vs. 102.13; *p* = 0.003), and SII (566.01 vs. 448.30; *p* = 0.011) were significantly elevated in the anemic group. MLR did not differ significantly between groups (0.48 vs. 0.42; *p* = 0.155), and absolute leukocyte and neutrophil counts were also comparable.

Multivariable logistic regression models were constructed to evaluate the independent association between inflammatory markers and anemia after adjustment for age, sex, BMI, type 2 diabetes mellitus, and eGFR. After standardization of continuous predictors, NLR (OR 4.09; 95% CI 1.50–11.19; *p* = 0.006), PLR (OR 4.89; 95% CI 1.41–17.02; *p* = 0.013), SII (OR 4.96; 95% CI 1.35–18.14; *p* = 0.016), and CRP (OR 2.17; 95% CI 1.07–4.39; *p* = 0.032) were independently associated with anemia. MLR was not significantly associated with anemia in the adjusted model (OR 1.84; 95% CI 0.91–3.74; *p* = 0.092). After Benjamini–Hochberg correction for multiple comparisons, the associations for NLR (*q* = 0.027), PLR (*q* = 0.027), SII (*q* = 0.027), and CRP (*q* = 0.040) all remained significant at FDR < 0.05, whereas MLR did not (*q* = 0.092), consistent with the uncorrected analysis. Results of the multivariable analysis are presented in Table 2.

**Figure 2:**
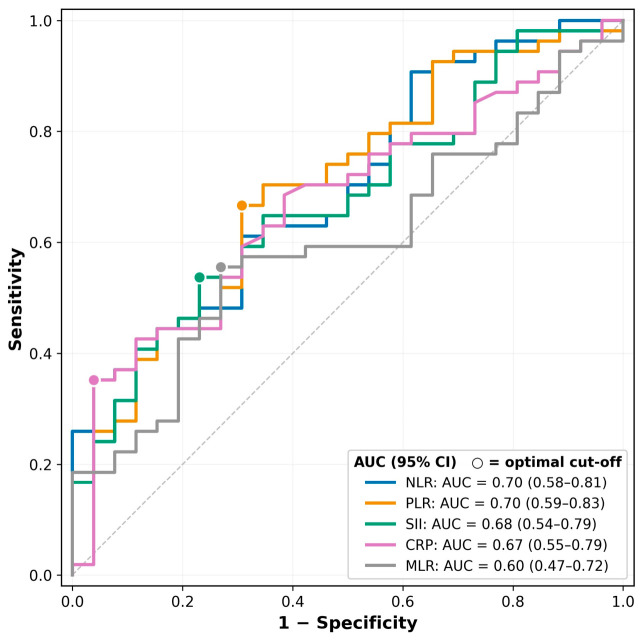
Receiver operating characteristic (ROC) curves for the five inflammatory markers (CRP, NLR, MLR, PLR, SII) in predicting anemia at entry into cardiac rehabilitation programme after TAVI. AUC values: NLR = 0.70, PLR = 0.70, SII = 0.68, CRP = 0.67, MLR = 0.60. AUC: area under the curve; CRP: C-reactive protein; MLR: monocyte-to-lymphocyte ratio; NLR: neutrophil-to-lymphocyte ratio; PLR: platelet-to-lymphocyte ratio; SII: systemic immune–inflammation index; TAVI: transcatheter aortic valve implantation.

Receiver operating characteristic (ROC) curve analysis demonstrated moderate discriminative performance for all significant markers: the area under the curve (AUC) was 0.70 for both NLR and PLR, 0.68 for SII, and 0.67 for CRP. MLR showed an AUC of 0.60 (Figure 2). At the optimal cut-off determined by the Youden index, NLR (cut-off 3.64) showed sensitivity 35.2% and specificity 96.2%; PLR (cut-off 122.1) showed sensitivity 66.7% and specificity 69.2%; SII (cut-off 560.5) showed sensitivity 53.7% and specificity 76.9%; CRP (cut-off 6.29 mg/L) showed sensitivity 35.2% and specificity 96.2%; and MLR (cut-off 0.481) showed sensitivity 55.6% and specificity 73.1% (Table 3).

To avoid conflating univariate ROC estimates with multivariable model diagnostics, calibration and internal validation metrics for the adjusted logistic regression models are presented separately in Table 4. These diagnostics correspond to the separate multivariable models, each adjusted for age, sex, BMI, type 2 diabetes mellitus, and eGFR.

## 4. Discussion

The aim of this study was to quantify the prevalence of anemia at entry into the cardiac rehabilitation programme following TAVI and to evaluate the independent association between systemic inflammatory burden and post-procedural anemia. In the analyzed cohort, anemia was present in approximately two-thirds of patients, defined using standard WHO thresholds widely adopted in clinical practice [20].

The high prevalence of anemia observed is consistent with prior studies reporting that anemia is common among patients with severe aortic stenosis undergoing TAVI, with reported rates ranging from 42% to 67% [11]. In a large multicenter observational study of 1696 TAVI patients, De Jaegere et al. found a preoperative anemia prevalence of 57%, with anemia independently associated with 1-year mortality [11]. A subsequent meta-analysis of 15 studies comprising 11,657 patients confirmed that baseline anemia was associated with significantly increased early and midterm mortality, with a graded inverse relationship between hemoglobin levels and survival [12]. More recently, Jiménez-Xarrié et al. reported baseline anemia rates ranging from 39% to 72% across 11 studies, with iron deficiency identified as the most common etiology in approximately 75% of cases [13]. The particularly high prevalence in our cohort (67.5%) may reflect the later assessment timepoint—entry into CR phase rather than the pre-procedural evaluation—as anemia may be amplified by the persistence of inflammation-mediated hematopoietic suppression.

A comprehensive review of blood disorders in TAVI patients highlighted the multifactorial nature of post-procedural hemoglobin decline, emphasizing that mechanisms extend well beyond overt procedural bleeding and include hemodilution from intravenous fluids and redistribution of interstitial fluids following acute hemodynamic stress [16]. In-hospital hemoglobin decline without overt bleeding has been independently associated with higher in-hospital mortality [21], supporting the concept that post-TAVI anemia is not merely a consequence of procedural blood loss but reflects a broader, multifactorial biological vulnerability that persists into the early post-procedural period—including at the time of entry into structured cardiac rehabilitation.

Our study revealed that, compared with non-anemic patients, those with anemia exhibited significantly higher levels of CRP as well as elevated NLR, PLR, and SII, suggesting a more pronounced inflammatory activation at the time of admission to the rehabilitation program. In multivariable logistic regression models adjusted for age, sex, BMI, type 2 diabetes mellitus, and eGFR, CRP, NLR, PLR, and SII remained independent predictors of anemia, whereas MLR did not reach statistical significance—indicating that not all leukocyte-derived ratios carry equivalent biological information in this context.

With regard to the SII specifically, our findings are contextualized in a growing body of evidence on this composite biomarker in the TAVI setting. Tosu et al. were among the first to demonstrate the prognostic value of SII in TAVI patients: in a retrospective cohort of 120 patients, SII was an independent predictor of MACEs—including stroke, re-hospitalization, and short-term mortality—at 6-month follow-up (HR 1.002; 95% CI 1.001–1.003; *p* < 0.01), outperforming CRP in multivariate analyses [7]. Ertem et al. extended this evidence by demonstrating that SII was an independent predictor of contrast-induced nephropathy (CIN) in 130 patients undergoing TAVR, with significantly higher SII values in patients who developed CIN compared with those who did not (1069 vs. 598; *p* = 0.003), highlighting the relevance of this marker for periprocedural complications beyond mortality [8]. A more recent retrospective cohort study of 138 TAVR patients, with a median follow-up of 12.5 months, further confirmed a significant positive linear association between SII and the risk of MACE (comprising all-cause death, cardiovascular death, non-fatal myocardial infarction, or non-fatal stroke), with this association maintained across multiple subgroups [9]. At the broader cardiovascular level, a systematic review and meta-analysis by Ye et al. encompassing multiple cardiovascular disease entities demonstrated that elevated SII is significantly associated with worse prognosis, positioning SII as a more comprehensive marker than individual ratios such as NLR or PLR alone [10]. The discriminative role of SII has also been documented beyond the TAVI population, across diverse cardiovascular conditions sharing an inflammatory substrate. Karakayali et al. demonstrated that SII was an independent predictor of the reverse-dipper circadian pattern in newly diagnosed hypertensive patients, with an optimal cut-off of 639.73 yielding a sensitivity of 63.3% and specificity of 84.2% (AUC 0.751) [22]. In a separate cohort, the same group reported that SII was independently associated with ischemia in patients with non-obstructive coronary arteries (INOCA), again with modest standalone discriminative performance (AUC 0.651; sensitivity 44.8%, specificity 78.8%) [23]. The discriminative performance reported in these studies (AUC 0.65–0.75) is closely concordant with the values observed in our cohort (AUC 0.67–0.70 univariate), reinforcing the notion that composite inflammatory indices such as SII capture a reproducible but moderate signal across distinct cardiovascular phenotypes—consistent with their value as accessible markers of biological vulnerability rather than standalone diagnostic tools. Of particular relevance to the present work, Altunova et al. observed that among STEMI patients undergoing primary percutaneous coronary intervention those in the higher-SII group exhibited significantly lower hemoglobin levels alongside greater coronary disease complexity (residual SYNTAX score; AUC 0.82, sensitivity 73.8%, specificity 72.3%) [24]. This inverse relationship between systemic inflammatory burden and hemoglobin, observed in an independent cardiovascular cohort, parallels the central association reported in our study and supports the biological coherence of an inflammation–anemia link across the cardiovascular disease spectrum.

For NLR and PLR, the literature has similarly focused on mortality and procedural complications. Rawat et al. published a systematic review and meta-analysis demonstrating that elevated NLR was significantly associated with increased mortality risk in TAVI patients (relative risk 1.37; 95% CI 1.08–1.74), with follow-up duration identified as a significant source of heterogeneity [5]. In a large registry of 1152 TAVI patients, Merdler et al. found that high NLR (above the median value of 4.1) was independently associated with 3-year mortality (HR 1.47; 95% CI 1.09–1.99; *p* = 0.013) [6]. Condado et al. demonstrated that NLR and PLR were predictive of 30-day early safety outcomes after TAVR and that a significant post-procedural increase in NLR was associated with worse 1-year survival [17]. Nair et al. showed that NLR was associated with 30-day mortality, while PLR had limited independent predictive value and STS-PROM score remained the strongest predictor of long-term survival [25]. Navani et al. found no significant association between preprocedural PLR and 30-day MACE in 470 patients [26], whereas Tosu et al. (2019) reported that elevated pre-procedural PLR was associated with vascular complications, stroke, and mid-term mortality during six-month follow-up in a cohort of 100 patients [27]. The discordance across these studies underscores the importance of using composite indices such as SII, which by integrating three cell lineages may capture more of the immune–inflammatory complexity than individual ratios.

We did not identify prior studies specifically investigating the relationship between composite inflammatory indices and anemia in post-TAVI patients at the specific timepoint of cardiac rehabilitation entry. However, converging evidence from multiple clinical settings supports the plausibility of this association. In a large population retrospective analysis of 14,261 subjects, Alshuweishi et al. reported that elevated NLR was independently associated with anemia risk (OR 1.91; 95% CI 1.47–2.48; *p* < 0.0001) alongside CRP, though with modest diagnostic performance on ROC analysis—consistent with our findings (AUC ≈ 0.70) [28]. Another population-based NHANES analysis similarly demonstrated that higher SII was independently associated with anemia (OR 1.51) in the general adult population [29], while data from healthy Danish blood donors linked low-grade CRP elevation to lower hemoglobin levels [30]. In oncologic populations, Zhang et al. identified elevated NLR as an independent predictor of anemia in 4,955 older patients with cancer (OR 1.97; 95% CI 1.73–2.24) [31], and evidence from chronic kidney disease cohorts consistently links NLR, PLR, and SII to anemia via hepcidin-mediated iron restriction [32]. In our cohort, renal function did not differ between anemic and non-anemic patients, suggesting that systemic inflammation—rather than renal insufficiency—may represent the dominant mechanistic pathway. Across all these settings, the magnitude of association observed in our post-TAVI population (OR ≈ 4–5) appears substantially higher, likely reflecting the compounded biological vulnerability of post-procedural, elderly cardiovascular patients. Unlike absolute leukocyte or neutrophil counts, which were comparable between groups, composite inflammatory ratios demonstrated consistent independent associations with anemia, supporting the concept that relative immune imbalance rather than crude inflammatory cell elevation underlies post-TAVI hematologic impairment.

A potentially distinctive feature of the present study is its focus on anemia—rather than mortality or adverse events—and specifically on the clinical timepoint of cardiac rehabilitation entry, a transitional period that has received limited dedicated attention in the literature. Evidence supports the safety and efficacy of exercise-based CR in TAVI patients, with documented improvements in exercise tolerance and functional independence [18,19]. Within this context, integrated assessment of inflammatory burden and hematologic status at CR admission may provide clinically actionable information for risk stratification and targeted diagnostic workup.

The observed association between systemic inflammatory burden and anemia may, hypothetically, reflect the “anemia of inflammation” phenotype—a mechanism in which pro-inflammatory signaling suppresses erythropoiesis and restricts iron availability through hepcidin-mediated pathways [14,15]. However, it must be emphasized that this mechanistic interpretation cannot be established from the present data. Our study lacks measurements of ferritin, transferrin saturation, soluble transferrin receptor, serum hepcidin, or pre-procedural hemoglobin values. Consequently, it is not possible to differentiate between absolute iron deficiency, functional iron deficiency, anemia of chronic disease, or mixed etiologies. The association observed between composite inflammatory indices and anemia is purely statistical and should not be interpreted as evidence of a specific pathophysiological mechanism. Although characterizing the detailed molecular pathway—including the IL-6-hepcidin-ferroportin-JAK2/STAT3 axis—would have been informative, the relevant mechanistic markers were not assessed in the present study; this pathway is therefore presented as biologically plausible background knowledge [14,15] rather than as a finding, and no mechanistic conclusions can be drawn from our data.

In addition to anemia, thrombocytopenia is another blood disorder known to be associated with inflammation. One study investigating patients undergoing TAVR with the Edwards SAPIEN valve demonstrated a clear association between systemic inflammation and post-procedural thrombocytopenia. Following valve deployment, platelet counts and platelet reactivity declined, while inflammatory biomarkers such as interleukin-6 and S100A8/A9 increased. Notably, perioperative elevations in inflammatory markers predicted subsequent declines in platelet count, and sustained thrombocytopenia was associated with increased post-procedural mortality. These findings further support the concept that TAVR induces a thromboinflammatory state, linking inflammatory activation to hematologic alterations and adverse outcomes [33].

The univariate AUC values for individual inflammatory markers (0.67–0.70) reflect fair-to-moderate discrimination and do not support standalone diagnostic use or immediate clinical implementation. We explicitly acknowledge that these values are insufficient to justify routine clinical application of these markers as anemia-screening tools. It is important to note, however, that these AUC values derive from univariate ROC analysis of individual markers; the bootstrap-corrected AUC values from the fully adjusted multivariable models ranged from 0.732 to 0.771 (Table 4), indicating meaningfully better discrimination when markers are considered in the context of relevant clinical covariates. Furthermore, the moderate univariate AUC is consistent with the expected performance of single inflammatory biomarkers in a multifactorial condition such as post-TAVI anemia, where etiology encompasses iron deficiency, chronic disease mechanisms, procedural factors, and nutritional deficiency simultaneously. A more comprehensive predictive model would require integration of iron metabolism parameters (ferritin, transferrin saturation), nutritional markers, and frailty indices—data which were not systematically available in the present cohort.

From a research perspective, our findings suggest that routinely available inflammatory indices—NLR, PLR, SII, and CRP—are associated with anemia at CR entry after TAVI and may represent accessible markers of biological vulnerability in this population. However, given the moderate discriminative performance (univariate AUC 0.67–0.70), these markers should not be interpreted as clinically validated diagnostic tools, and their routine implementation cannot be recommended on the basis of the present data alone. Rather, these results provide a hypothesis-generating foundation to support future prospective studies that incorporate comprehensive iron metabolism profiling and formal clinical decision modelling, with the aim of developing validated, actionable risk stratification tools for the post-TAVI rehabilitation setting.

A broader pathophysiological consideration further enriches the interpretation of our findings. Anemia is frequently underrecognized during the workup of aortic stenosis, yet it bears a clinically meaningful, bidirectional relationship with valve disease. Because cardiac output rises in the anemic state and the transvalvular gradient increases with transvalvular flow—such that the calculated valve area is inversely related to the square root of the gradient—an anemia-driven increase in cardiac output can inflate the measured gradient and thereby exaggerate the apparent hemodynamic severity of stenosis, in some patients producing or aggravating symptoms attributable less to the valve itself than to the reduced oxygen-carrying capacity. Moreover, severe aortic stenosis can directly cause anemia: in Heyde’s syndrome, the high shear stress generated across the stenotic valve induces proteolytic cleavage of high-molecular-weight von Willebrand factor multimers, producing an acquired type 2A von Willebrand syndrome that predisposes to bleeding from gastrointestinal angiodysplasia and consequent chronic anemia [34,35]. This process carries inflammatory and angiogenic components—including VEGF-mediated angiodysplasia driven by reduced pulsatile flow—and characteristically improves after valve replacement, rendering it directly pertinent to a post-TAVI rehabilitation population. These mechanisms raise the possibility that, in a subset of our patients, the inflammatory burden and anemia we observed reflect not merely a non-specific comorbidity but a shared disease process intrinsic to the aortic valve pathology that prompted intervention. It is also relevant that the inflammatory consequences of TAVI itself are variable: an uncomplicated procedure may leave systemic inflammatory status largely unchanged, whereas peri-procedural embolism or microembolism can elevate inflammatory markers, adding a further layer to the interpretation of post-procedural measurements.

From a practical standpoint, these considerations argue for a structured diagnostic approach to anemia in this population—ideally initiated during the preprocedural evaluation of aortic stenosis rather than deferred to the rehabilitation phase. Because anemia is frequently overlooked amid the urgency of addressing a rising transvalvular gradient, systematically characterizing it before valve implantation could identify patients in whom anemia itself contributes to symptom burden and to the apparent hemodynamic severity of stenosis and could uncover an occult disease process such as Heyde’s syndrome. The same workup remains valuable in the post-TAVI rehabilitation setting, both to reassess anemia that persists after valve replacement and to detect procedure-related contributors. A pragmatic evaluation would include iron studies (ferritin, transferrin saturation, and soluble transferrin receptor) to distinguish absolute from functional iron deficiency and anemia of inflammation; reticulocyte count, haptoglobin, lactate dehydrogenase, and peripheral smear to detect ongoing hemolysis; vitamin B12 and folate to exclude nutritional contributors; and, where Heyde’s syndrome is suspected, assessment of von Willebrand factor multimers (or a platelet function screen such as the closure time) together with fecal occult blood testing and, if indicated, endoscopic evaluation for gastrointestinal angiodysplasia. Framing anemia as a diagnostic priority both before and after valve intervention—rather than an incidental finding—may reveal reversible contributors and refine the attribution of symptoms between the valve and the hematologic milieu.

### Limitations

The results should be interpreted in light of several limitations inherent to the retrospective, single-center observational design. Accordingly, the observed associations do not establish causality. Iron metabolism parameters, including ferritin, transferrin saturation, soluble transferrin receptor, and hepcidin, were not available; therefore, the precise etiology of anemia could not be determined, and mechanistic interpretations remain hypothesis-generating.

Preprocedural hemoglobin values were also not systematically available, preventing assessment of whether anemia at cardiac rehabilitation entry reflected pre-existing anemia, newly developed anemia, or periprocedural hemoglobin decline. However, defining hemoglobin trajectories was not the primary objective of the present study. Rather, our aim was to characterize the inflammatory and hematologic profile of patients at the clinically relevant time point of admission to cardiac rehabilitation after TAVI, when risk stratification and individualized recovery planning are performed.

The exclusion of patients with VARC-2 bleeding, transfusion, or vascular complications may limit generalizability to the broader unselected post-TAVI population. However, this was a deliberate methodological decision aimed at reducing major confounding. These events may cause acute hemoglobin decline through direct blood loss, hemodilution, or vascular injury, while also independently increasing inflammatory markers through tissue injury, hematoma formation, transfusion-related immune activation, prolonged hospitalization, or procedural stress. Including such patients would therefore have created a circular confounding pathway in which the same procedural complication could contribute simultaneously to anemia and systemic inflammation.

Finally, although the adjustment model included clinically and biologically relevant covariates, other factors such as frailty, nutritional status, nutritional deficiencies, and procedural characteristics were not available in this retrospective dataset. Therefore, the findings should be regarded as hypothesis-generating and require validation in larger, prospective, multicenter studies.

## 5. Conclusions

In patients entering cardiac rehabilitation after TAVI, anemia is highly prevalent (67.5%) and is independently associated with systemic inflammatory burden, as assessed by CRP, NLR, PLR, and SII. The SII emerged as the marker with the strongest adjusted association with anemia in this cohort. The clinical relevance of these findings lies in identifying a readily assessable inflammatory signature at CR entry that may help stratify patients with greater biological vulnerability, warranting further targeted diagnostic evaluation. Given the moderate discriminative performance of individual markers (univariate AUC 0.67–0.70), these findings do not support immediate clinical implementation and should be regarded as hypothesis-generating. Their potential value lies in motivating future prospective studies that incorporate comprehensive iron metabolism profiling, frailty assessment, and formal clinical decision modelling to develop validated risk stratification tools for the post-TAVI rehabilitation setting. Future prospective, multicenter studies incorporating comprehensive iron metabolism profiling, nutritional assessment, and longitudinal inflammatory trajectories are needed to clarify the mechanistic basis of the observed association.

## Figures and Tables

**Table 1:** Baseline clinical and laboratory characteristics of the study population, stratified by anemia status.

Variable	All Patients (*n* = 80)	No Anemia (*n* = 26)	Anemia (*n* = 54)	*p*-Value
Age (years) †	75.0 (6.0)	75.0 (7.5)	77.0 (6.0)	0.362
Female sex □	49 (61.3%)	16 (61.5%)	33 (61.1%)	1.000
BMI (kg/m^2^) †	29.34 (6.08)	30.44 (6.18)	27.70 (6.39)	0.012
Type 2 diabetes □	36 (45.0%)	7 (26.9%)	29 (53.7%)	0.044
Hypertension □	60 (75.0%)	17 (65.4%)	43 (79.6%)	0.270
Current/recent smoker □	17 (21.2%)	4 (15.4%)	13 (24.1%)	0.690
Cancer history □	6 (7.5%)	2 (7.7%)	4 (7.4%)	1.000
COPD □	13 (16.2%)	5 (19.2%)	8 (14.8%)	0.859
Prior stroke □	9 (11.2%)	4 (15.4%)	5 (9.3%)	0.664
Prior angioplasty □	22 (27.5%)	4 (15.4%)	18 (33.3%)	0.157
Atrial fibrillation □	28 (35.0%)	11 (42.3%)	17 (31.5%)	0.480
Pacemaker □	12 (15.0%)	3 (11.5%)	9 (16.7%)	0.742
LVEF-Preserved □	70 (87.5%)	24 (92.3%)	46 (85.2%)	
LVEF-Mildly reduced □	3 (3.8%)	1 (3.8%)	2 (3.7%)	
LVEF-Reduced □	7 (8.7%)	1 (3.8%)	6 (11.1%)	0.560
Systolic BP (mmHg) †	130.0 (20.0)	138.5 (17.0)	130.0 (23.0)	0.035
Diastolic BP (mmHg) †	70.0 (15.0)	76.0 (11.25)	70.0 (14.5)	0.012
Heart rate (bpm) †	75.0 (21.0)	75.5 (14.25)	75.0 (22.0)	0.617
eGFR (mL/min/1.73m^2^) †	53.72 (15.34)	53.72 (13.88)	52.50 (18.20)	0.849
Hemoglobin (g/dL) †	11.30 (2.18)	13.25 (1.17)	10.80 (1.28)	<0.001
Leukocyte count (×10^9^/L) †	6.67 (2.11)	6.89 (1.78)	6.40 (2.42)	0.692
Neutrophil count (×10^9^/L) †	4.06 (1.46)	4.04 (1.06)	4.21 (1.76)	0.450
CRP (mg/L) †	3.53 (4.30)	2.58 (3.05)	4.25 (5.93)	0.014
NLR †	2.61 (1.52)	2.34 (1.27)	2.86 (2.03)	0.004
MLR †	0.44 (0.25)	0.42 (0.16)	0.48 (0.24)	0.155
PLR †	125.93 (68.05)	102.13 (61.56)	132.33 (70.54)	0.003
SII †	520.61 (335.90)	448.30 (267.31)	566.01 (414.32)	0.011

† Data are presented as median (interquartile width). □ Data are presented as number (percentage). Continuous variables: Mann–Whitney U test. Categorical variables: chi-square test or Fisher’s exact test. BMI: body mass index; BP: blood pressure; COPD: chronic obstructive pulmonary disease; CRP: C-reactive protein; eGFR: estimated glomerular filtration rate; LVEF: left ventricular ejection fraction; MLR: monocyte-to-lymphocyte ratio; NLR: neutrophil-to-lymphocyte ratio; PLR: platelet-to-lymphocyte ratio; SII: systemic immune–inflammation index.

**Table 2:** Multivariable logistic regression analysis for the association between inflammatory markers and anemia.

Marker	Adjusted OR *	95% Confidence Interval	*p*-Value
CRP	2.17	1.07–4.39	0.032
NLR	4.09	1.50–11.19	0.006
MLR	1.84	0.91–3.74	0.092
PLR	4.89	1.41–17.02	0.013
SII	4.96	1.35–18.14	0.016

* Odds ratios expressed per 1 standard deviation increase. Model adjusted for age, sex, BMI, type 2 diabetes mellitus, and eGFR. CRP: C-reactive protein; MLR: monocyte-to-lymphocyte ratio; NLR: neutrophil-to-lymphocyte ratio; PLR: platelet-to-lymphocyte ratio; SII: systemic immune–inflammation index.

**Table 3:** Univariate ROC analysis and diagnostic performance of inflammatory markers for anemia status at cardiac rehabilitation entry after TAVI.

Marker	AUC	95% CI	Cut-Off (Youden)	Sensitivity	Specificity
NLR	0.70	0.58–0.81	3.64	35.2%	96.2%
PLR	0.70	0.59–0.83	122.1	66.7%	69.2%
SII	0.68	0.54–0.79	560.5	53.7%	76.9%
CRP	0.67	0.55–0.79	6.29 mg/L	35.2%	96.2%
MLR	0.60	0.47–0.72	0.481	55.6%	73.1%

AUC: area under the curve; CI: confidence interval; CRP: C-reactive protein; MLR: monocyte-to-lymphocyte ratio; NLR: neutrophil-to-lymphocyte ratio; PLR: platelet-to-lymphocyte ratio; SII: systemic immune–inflammation index. Cut-off values, sensitivity, and specificity were determined by the Youden index from univariate ROC analysis. The 95% CI for the AUC was estimated via 1000-iteration bootstrap resampling.

**Table 4:** Calibration and internal validation of the adjusted multivariable logistic regression models.

Marker Included in Adjusted Model	Bootstrap-Corrected Adjusted AUC	Optimism	HL *p*-Value
NLR	0.771	0.052	0.568
PLR	0.737	0.060	0.648
SII	0.752	0.055	0.976
CRP	0.732	0.059	0.672
MLR	0.719	0.063	0.810

AUC: area under the curve; CRP: C-reactive protein; HL: Hosmer–Lemeshow; MLR: monocyte-to-lymphocyte ratio; NLR: neutrophil-to-lymphocyte ratio; PLR: platelet-to-lymphocyte ratio; SII: systemic immune–inflammation index. Bootstrap-corrected adjusted AUC values were derived from 1000-iteration bootstrap resampling of the multivariable logistic regression models adjusted for age, sex, BMI, type 2 diabetes mellitus, and eGFR. HL *p*-value refers to the Hosmer–Lemeshow goodness-of-fit test; values > 0.05 indicate no evidence of poor calibration.

## Data Availability

The data presented in this study are available on request from the corresponding author due to privacy and ethical restrictions.

## Abbreviations

The following abbreviations are used in this manuscript:

AUC	Area under the curve
BMI	Body mass index
BP	Blood pressure
CI	Confidence interval
COPD	Chronic obstructive pulmonary disease
CR	Cardiac rehabilitation
CRP	C-reactive protein
eGFR	Estimated glomerular filtration rate
HR	Hazard ratio
IL-6	Interleukin-6
IQW	Interquartile width
LVEF	Left ventricular ejection fraction
MACE	Major adverse cardiovascular events
MLR	Monocyte-to-lymphocyte ratio
NLR	Neutrophil-to-lymphocyte ratio
OR	Odds ratio
PLR	Platelet-to-lymphocyte ratio
ROC	Receiver operating characteristic
SII	Systemic immune–inflammation index
SIRS	Systemic inflammatory response syndrome
STS-PROM	Society of Thoracic Surgeons Predicted Risk of Mortality
TAVI	Transcatheter aortic valve implantation
TAVR	Transcatheter aortic valve replacement
WHO	World Health Organization
